# Haploinsufficiency of the E3 Ubiquitin Ligase C-Terminus of Heat Shock Cognate 70 Interacting Protein (CHIP) Produces Specific Behavioral Impairments

**DOI:** 10.1371/journal.pone.0036340

**Published:** 2012-05-11

**Authors:** BethAnn McLaughlin, Matthew A. Buendia, Tommy P. Saborido, Amy M. Palubinsky, Jeannette N. Stankowski, Gregg D. Stanwood

**Affiliations:** 1 Department of Neurology, Vanderbilt University School of Medicine, Nashville, Tennessee, United States of America; 2 Department of Pharmacology, Vanderbilt University School of Medicine, Nashville, Tennessee, United States of America; 3 Vanderbilt Kennedy Center for Research on Human Development, Vanderbilt University School of Medicine, Nashville, Tennessee, United States of America; 4 Neuroscience Graduate Group, Vanderbilt University School of Medicine, Nashville, Tennessee, United States of America; Hertie Institute for Clinical Brain Research and German Center for Neurodegenerative Diseases, Germany

## Abstract

The multifunctional E3 ubiquitin ligase CHIP is an essential interacting partner of HSP70, which together promote the proteasomal degradation of client proteins. Acute CHIP overexpression provides neuroprotection against neurotoxic mitochondrial stress, glucocorticoids, and accumulation of toxic amyloid fragments, as well as genetic mutations in other E3 ligases, which have been shown to result in familial Parkinson's disease. These studies have created a great deal of interest in understanding CHIP activity, expression and modulation. While *CHIP* knockout mice have the potential to provide essential insights into the molecular control of cell fate and survival, the animals have been difficult to characterize *in vivo* due to severe phenotypic and behavioral dysfunction, which have thus far been poorly characterized. Therefore, in the present study we conducted a battery of neurobehavioral and physiological assays of adult *CHIP* heterozygotic (HET) mutant mice to provide a better understanding of the functional consequence of *CHIP* deficiency. We found that *CHIP* HET mice had normal body and brain weight, body temperature, muscle tone and breathing patterns, but do have a significant elevation in baseline heart rate. Meanwhile basic behavioral screens of sensory, motor, emotional and cognitive functions were normative. We observed no alterations in performance in the elevated plus maze, light-dark preference and tail suspension assays, or two simple cognitive tasks: novel object recognition and spontaneous alternation in a Y maze. Significant deficits were found, however, when *CHIP* HET mice performed wire hang, inverted screen, wire maneuver, and open field tasks. Taken together, our data indicate a clear subset of behaviors that are altered at baseline in *CHIP* deficient animals, which will further guide whole animal studies of the effects of CHIP dysregulation on cardiac function, brain circuitry and function, and responsiveness to environmental and cellular stress.

## Introduction

Heat shock proteins (HSPs) are highly conserved, abundantly expressed chaperone proteins with diverse functions, including the assembly of multi-protein complexes, transport of nascent polypeptides, regulation of protein folding, and neuroprotection [1]. Heat shock protein 70 (HSP70) is the major stress-inducible chaperone in the CNS and is upregulated with thermal stress, oxidative injury, and after various acute and chronic insults [2], [3], [4]. HSP70 operates as part of a multi-protein complex in which associated co-chaperone molecules can alter client proteins bound to HSP70 thereby affecting the trafficking, degradation and refolding activities of HSP70 [5], [6], [7]. The multifunctional E3 ubiquitin ligase, C-terminus of heat shock cognate 70 interacting protein (CHIP) is a key interacting protein of HSP70, and appears to primarily promote client protein ubiquitination and subsequent proteasomal degradation [5], [6].

Over 30 proteins have been identified as specific substrates of CHIP, many of which are associated with cellular differentiation and survival, as well as susceptibility to stress [5]. CHIP is a central modulator of both the activity and expression of HSP70 as it interacts with the chaperone and also promotes its degradation [8]. Other CHIP client proteins have central roles in glucocorticoid response, tau degradation, and both p53 and cAMP signaling [9], [10], [11], [12], [13], [14]. Loss of CHIP might result in increased susceptibility to stress and increased cell death due to a dysregulation of essential client proteins. Indeed, overexpression of CHIP can decrease central nervous system cell death in models of chronic neurodegenerative diseases, including Parkinson's and Alzheimer's disease [11], [15], [16]. Our group has, however, recently shown that CHIP can affect essential signaling pathways and be deleterious to cellular health when chronically overexpressed [17], suggesting a delicate balance must exist to promote cell survival.

In order to better understand the role of CHIP as a mediator of the chaperone response, mice that are deficient in *CHIP* expression were developed by Patterson and colleagues [18], [19]. Knockout mice weigh 30% less than wild type (WT) mice at 3 and 12 months of age, have increased and accelerated skeletal muscle atrophy, and possess significantly decreased body fat stores at 12 months. The authors also found that *CHIP* deficient mice have larger hearts at 12 months of age, indicative of cardiac hypertrophy. Together with the fact that *CHIP* knockout animals exhibit signs of osteoporosis and kyphosis, these data suggest an advanced-ageing phenotype and also help to explain the significantly decreased lifespan of these animals [18], [19].

We hypothesize that cellular and circuit adaptations and maladaptations to CHIP or other chaperone molecules contributes to individual vulnerabilities that may result in the development of neurodegenerative disorders and/or increased risk for neurological dysfunction following ischemic events [20], [21]. While the role of oxidative and nitrosylative stress and CNS degeneration is well appreciated [22], these pathways may also contribute to the more subtle manifestations of diseases like Parkinson's and Alzheimer's which have an affective component including comorbid depression. Indeed, links between cellular stress responses and vulnerability to stress-related mental health disorders has also been recently postulated [23], [24].

To test this hypothesis we examine the effects of *CHIP* haploinsufficiency on behavioral and neurological function in mice similar to previous studies of other large impact brain mutations, such as those in *Reelin* [25], [26], *Scrapper* [27], and monoamine transporters [28]. In this work, we report the presence of specific motor disturbances in *CHIP* heterozygous (HET) mice in the absence of changes in emotional reactivity, learning and memory, or behavioral despair.

## Materials and Methods

### Animals and General Behavioral Methods

*CHIP* (also known as *Stub1*) heterozygous and wild type littermate mice were bred in house from matings of heterozygous to wild type mice, and weaned at postnatal day (P) 21 at which time genotypes were determined by PCR analysis of tail tissue using standard procedures [18], [19]. Mice were housed 2–5 per cage in a temperature and humidity controlled environment (lights on 0600-1800 h) within AAALAC approved Vanderbilt University Animal Care Facilities. Food and water were available *ad libitum*. All procedures were approved by the Animal Care and Use Committee at Vanderbilt University. Mice were extensively handled for at least one week prior to the beginning of experiments, were at least P70 prior to testing, and were habituated to the testing rooms for ∼30 min prior to testing. Only male mice were tested. Testing apparatuses were cleaned with Vimoba spray (Quip Labs, Wilmington, DE) between animals and wiped with paper towels to clear the solution and any debris. Mice were weighed prior to and at the conclusion of behavioral testing (no significant changes). After behavioral testing, mice were euthanized with an overdose of isoflurane and brains were removed and weighed.

### Wire Hang

Gross motor function was examined in a basic wire hang test. Mice were allowed to hang via their forepaws on a plastic wire (14 gauge) suspended 35 cm above the surface. Mice were able to use their back paws for balance and support during the test. Latency to fall into an arena with fresh bedding was recorded for up to 180 sec, at which point mice were removed from the wire and returned to the home cage.

### Inverted Screen

For the inverted screen test, 2–4 littermates were placed on a metal grid screen (10 cm×14 cm) with separate compartments. After placement, the grid was inverted 60 cm over a plastic cage containing fresh bedding. Latency to fall was recorded for up to 60 sec, at which point mice were removed from the apparatus and returned to the home cage. Two trials were conducted on separate days, and data averaged together.

### Grip Strength

Grip force was measured using a force gauge (San Diego Instruments, San Diego, CA) [29]. Five trials were carried out consecutively. Testing was conducted in normal lighting conditions (250–300 lux). Grip strength was recorded via a digital gauge that locked in the peak reading of each trial. The average grip strength score, given in Newtons, is the average of five trials.

### Rotarod

Motor coordination and balance were measured using a commercially available accelerating rotarod apparatus (Ugo Basile model 7650) as previously described [30]. Mice were placed on the rotating cylinder (3 cm in diameter) and confined to a section approximately 6.0 cm long by gray plastic dividers. The rotational speed of the cylinder was increased from 5 to 40 r.p.m. over a 5 min period. Latency at which mice fell off the rotating cylinder was measured. Each mouse was given three trials per day over a period of 3 days.

### Cutaneous Mechanical Sensitivity

Mice were lowered by their tails into a small box with wire mesh flooring, which is raised approximately 45 cm above the table surface. Von Frey filaments (North Coast Medical, Inc., Morgan Hill, CA) were applied perpendicularly against the planar surface of a back paw until bowing of the fiber occurred in ascending order of thickness. The bent filament was held for 2 sec and applied five times at 5 sec intervals. The test ended when the mouse performed three withdrawal responses out of five applications of the filament, with a withdrawal response being qualified as the paw being entirely removed from the mesh flooring in a motion not related to general locomotion.

### Elevated Plus Maze (EPM)

Anxiety responses were examined using a custom EPM (White, 67 cm length×6.5 cm width×15 cm height in the closed arms). The entire apparatus was elevated 40 cm from the ground and testing was conducted under normal lighting (250–300 lux). At the start of the 5 min trial, each mouse was lowered, by its tail, onto an open section of the maze, directly next to and facing a closed section. Sessions were recorded by a ceiling-mounted video camera connected to a computer using video acquisition and analysis software (ANY-maze, Stoelting, Wood Dale, IL). Data analyzed included % open arm time, number of zone crossings and distance traveled throughout the maze [31], [32].

### Y maze

Spontaneous alternation was examined using a 3-arm Y maze (6×35.5×10 cm) under normal lighting (250–300 lux). At the start of the 6 min trial, each mouse was lowered, by its tail, into one arm of the maze, facing towards the center junction. Sessions were recorded by a ceiling-mounted video camera connected to a computer using video acquisition and ANY-maze analysis software (Stoelting, Wood Dale, IL). Data analyzed included number of arm entries and sequence of entries, allowing calculation of % spontaneous alternation (consecutive entry into each of the three arms), as described previously [32], [33].

### Open Field Activity

Locomotor activity in a novel open field was measured using commercial open field activity chambers (Med Associates, 27×27×20.5 cm) that were contained within a light- and air-controlled environment (Med Associates, 64×45×42 cm). Location and movement were detected by the interruption of infrared beams by the body of the mouse (16 photocells in each horizontal direction, as well as 16 photocells elevated 4 cm to measure rearing) and were measured by the Med Associates Activity Monitor program [34]. Activity was measured for a 90 min session.

### Light-Dark Preference

The light-dark preference test uses an opaque insert placed within one-half of the Med Associates Open Field chambers. The dark insert measures 27 cm in length, 14 cm in width, and 20.5 cm in height, with a single door for entering/exiting a darkened area and is placed on the left side of the activity chamber. Movement and location are again detected by the interruption of infrared beams during a 10 min session.

### Novel Object Recognition

Nonspatial working memory was assessed in a two object recognition task [32], [33]. The day before testing, mice were habituated to the novel object arena (39.5×28.5×19.5 cm, Allentown, Inc., Allentown, NJ) for 20 min. On the test day, mice were placed into the cage with two identical objects (either 50 ml conical tubes weighted with sand or small blue microscope slide boxes on their side) placed on the two sides of the cage for 5 min. After a 10 min delay in their home cage they were returned to the arena and allowed to explore two objects, one identical to the original objects and the other different, for an additional 5 min. Time spent exploring novel versus familiar objects was recorded by post-hoc video analysis. A counter-balanced design was used to control for side of presentation and which of the pair of objects was novel. Exploration of the two identical objects was compared to verify that there was not a measurable object or side bias despite the experimental design.

### Tail Suspension Test (TST)

Behavioral despair was measured by suspending mice for 7 min by the tail from a vertical aluminum bar attached to the top of a box-like enclosure (Med Associates, 33×33×32 cm) that is open in the front. Mice are attached to the bar by tape placed ∼1.5 cm from the tip of the tail. Force transducers and automated software (Med Associates) is used to measure immobility. Settings utilized were a lower threshold of 7, upper threshold of 20, gain of 8, and resolution of 220 ms.

### Modified Irwin Screen and Miscellaneous Tests

Mice were screened for basic sensorimotor functions and reflexive measures in a modified Irwin battery [35], with minor modifications as previously described [36]. Oxygen saturation, heart rate and breath rate were measured in awake mice with a MouseOx system (Starr Life Sciences, Oakmont, PA) and CollarClip sensor (size small).

### Statistical Analysis

Genotype differences were assessed by unpaired Student's *t*-test or ANOVA as appropriate with significance defined as two-tailed p<0.05. Analyses of wire maneuver performance and von Frey filament required to produce a withdrawal response were analyzed by chi square (χ^2^).

## Results

### General Physiological Variables

**Figure 1:**
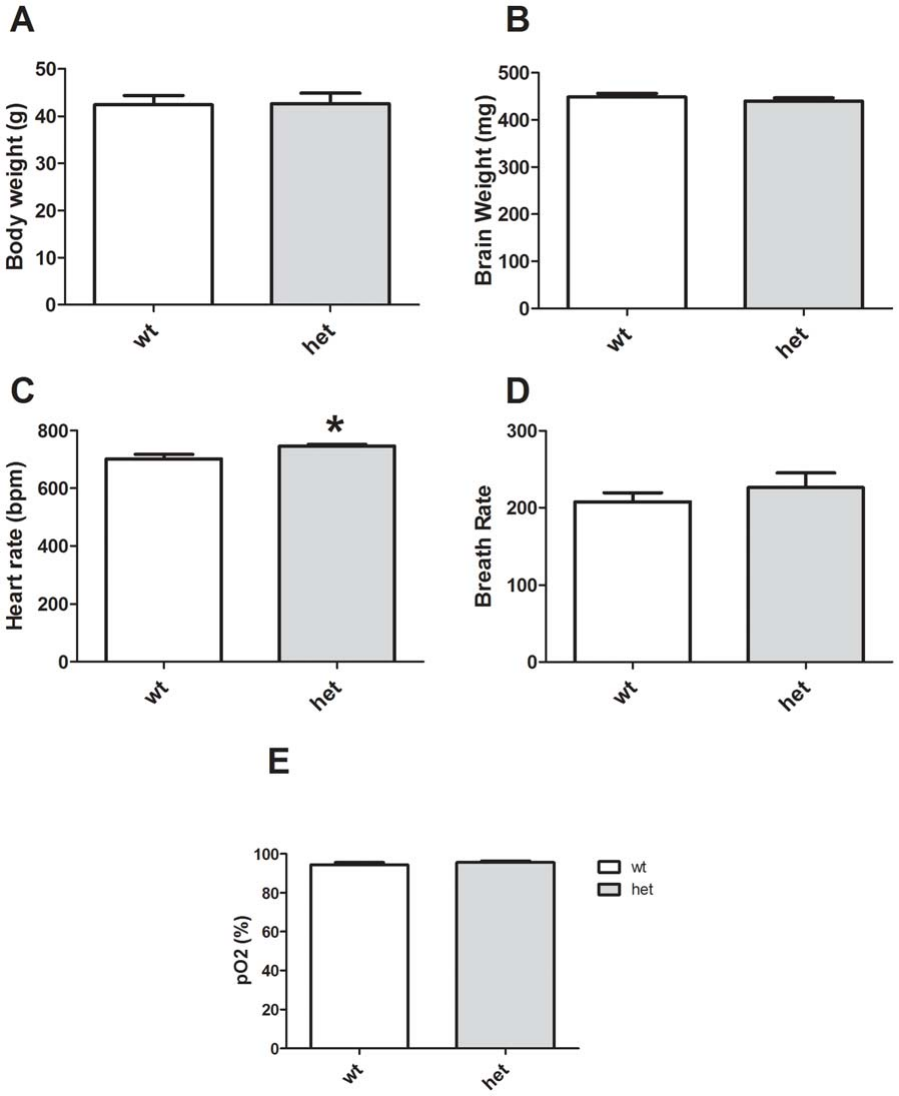
Specific physiological alterations in *CHIP* HET mice. Body weights (A) and brain weights (B) do not differ between *CHIP* WT and HET mice. (C) HET mice exhibit increased heart rate, but breath rate (D) and pO_2_ (E) are not significantly different from WT mice. *p<0.05.

Although *CHIP* knockout mice exhibit significant weight loss (30% or more), muscle atrophy, bone abnormalities and cardiac hypertrophy [12], [13] (and unpublished observations), HET mice have no obviously clear phenotype upon simple observation. Body and brain weights of *CHIP* HET mice did not differ from WT littermate controls (Figure 1A, *p* = 0.944 and 1B *p* = 0.386). There was a small but significant increase in the heart rate of HET mice (Figure 1C, *p* = 0.010); however, there were no significant differences in breath rate (Figure 1D, *p* = 0.459) or blood oxygenation (Figure 1E, *p* = 0.334) between genotypes. Mice were tested for additional physiological and reflexive measures using a modified Irwin battery, which indicated that the WT and HET genotypes were similar in most measures (Table 1).

**Table 1:** Modified Irwin Screen.

*Physical Condition*	+/+	+/−
Initial Body Weight (g)	33.4±1.95	34.8±2.07
Final Body Weights (g)	42.4±1.95	42.7±2.25
Final Brain Weight (mg)	447±7.05	442±6.80
Rectal Temperature (°C)	34.7±0.28	35.1±0.29
Normal Presence of Whiskers (%)	100	100
Well groomed (%)	100	100
Piloerection (%)	0	0
Fur missing on face (%)	0	0
Fur missing on body (%)	0	0
Wounds (%)	0	0
Agouti Fur Color (%)	100	100

*Behavior in novel environment*	+/+	+/−
Normal transfer behavior (%)	100	100
Normal body positioning (%)	100	100
Spontaneous Activity (%)		
Casual scratch, groom, slow movement	73	75
Vigorous scratch, groom, moderate movement	27	17
Vigorous scratch, rapid/dart movement	0	8
Normal respiration rate (%)	100	100
Tremors (%)	0	0
Palpebral closure (%)	100	100
Piloerection (%)	0	0
Normal gait (%)	100	100
Normal pelvic elevation (%)	100	100
Horizonal tail elevation (%)	100	100

*Reflexes or Reaction to Stimuli*	+/+	+/−
Touch escape (%)		
No response	55	67
Mild (escape response to firm stroke)	45	33
Moderate (escape response to light stroke)	0	0
Vigorous (escape response to approach)	0	0
Positional passivity - Does not struggle (%)	100	100
Trunk curl Present (%)	100	100
Reaching reflex before vibrissae contact (%)	100	100
Normal body tone (%)	100	100
Normal righting reflex (%)	100	100
Inverted Screen latency to fall (s) (see also Figure 2)	43.2±4.3	29.0±4.7*
Wire hang latency to fall (s) (see also Figure 2)	40.7±9.1	14.4±5.4*
Grip Strength (%)		
Slight grip, semi-effective	91	100
Moderate grip, effective	8	0
***Wire maneuver (%)***		* ***p<0.0001***
Active grip with hindlegs	72	8
Difficultly to grasp with hindlegs	18	17
Unable to grasp with hindlegs	9	25
Unable to lift hindlegs, falls within seconds	0	42
Falls immediately	0	8
Pole Climb - completes within 30 seconds	100	100

Mice were screened in a battery of measures to assess overall health and basic sensory and reflexive function. Values reflect the mean ± SEM of the percentage of animals exhibiting each attribute unless noted. Sample size: wild type (WT, +/+) n = 11, heterozygous (HET, +/−) n = 12.

* *p<0.05.*

### Simple Elicited Sensory and Motor Functions

**Figure 2:**
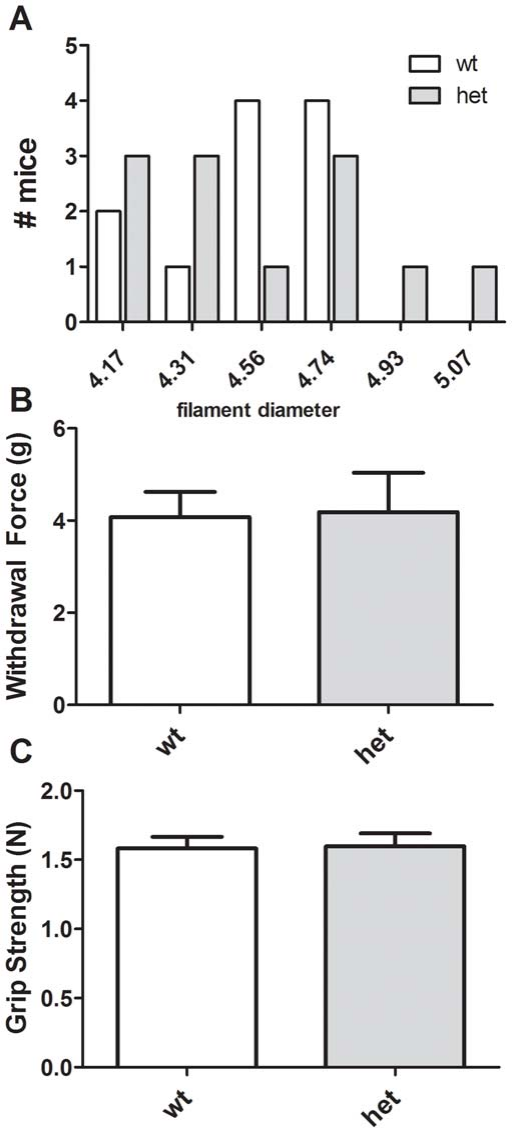
Normal somatosensory function and grip strength in *CHIP* HET mice. *CHIP* WT and HET mice exhibit normal paw withdrawal sensitivities to mechanical stimuli using von Frey filaments (A, B) and forepaw grip strength (C).

Paw withdrawal responses to somatosensory stimuli were examined using von Frey filaments. HET and WT mice did not differ either when analyzed by distribution of required filament to produce a response (Figure 2A, χ^2^ (5, *N* = 23, 5.11, *p* = 0.403), or by mean withdrawal force required (Figure 2B, *p* = 0.916). Muscle tone was measured using a grip strength apparatus and again no differences were revealed (Figure 2C, *p* = 0.908).

**Figure 3:**
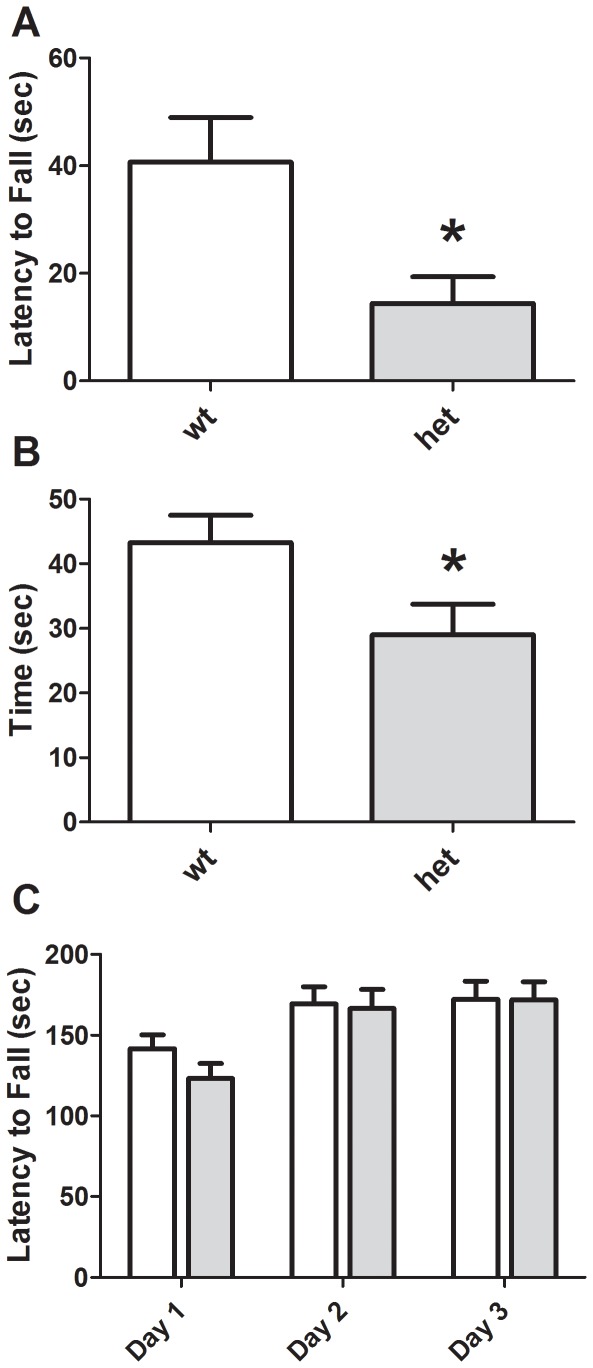
Motor abnormalities in *CHIP* HET mice. *CHIP* HET mice exhibit a reduced latency to fall from a wire hang apparatus (A) and reduced time holding onto an inverted screen (B), but normal performance and learning on an accelerating rotarod (C). *p<0.05.

*CHIP* HET mice exhibited a more rapid latency to fall from a wire hang test (Figure 3A and Table 1, *p* = 0.012) and inverted screen (Figure 3B and Table 1, *p* = 0.037), suggesting motor impairments. Success in a wire maneuver task was also impaired in *CHIP* HET mice (Table 1, χ^2^ [4, *N* = 23, 108.8, *p*<0.0001]). Motor learning, as assessed on an accelerating rotarod, was intact (Figure 3C). Two-way ANOVA revealed a significant effect of trial day, demonstrating the presence of motor learning [*F* (2, 67) = 3.27, *p*<0.05]. However, there were no significant differences based on genotype [*F* (1, 22) = 0.270, *p* = 0.6050] or genotype×trial day interaction [*p* = 0.846].

### Motivated and Emotional Behaviors

**Figure 4:**
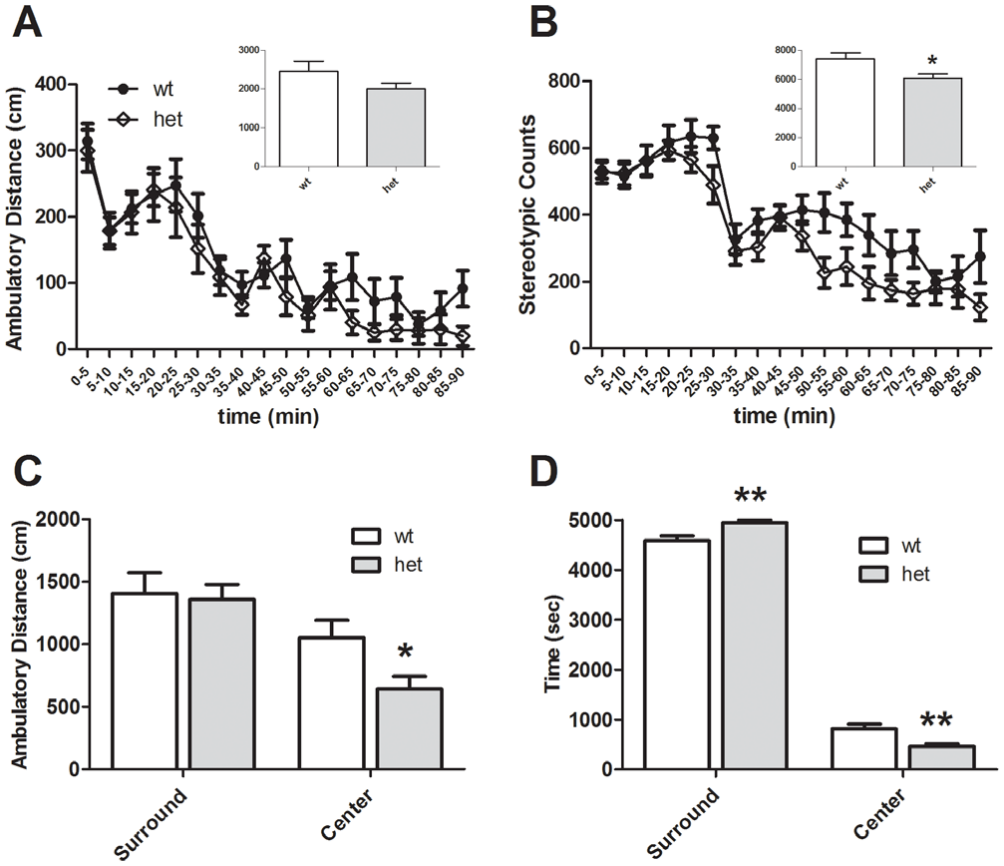
Open Field behaviors in *CHIP* WT and HET mice. *CHIP* HET mice exhibit a significant decrease in motor stereotypies within an open field apparatus (B), but only a non-significant trend in total ambulatory distance (panel A, p = .135). However, upon separation of the open field into center and surround zones, there is a significant decrease in distance (C) and time spent in the center (D), suggesting an increase in anxiety-related behavior. *p<0.05, **p<0.01.

Mice were exposed to a novel open field and locomotor exploration was measured over a 90 min period. HET and WT mice did not significantly differ in ambulatory distance (Figure 4A, *p* = 0.135) or time (data not shown), but *CHIP* HET mice did exhibit a small reduction in motor stereotypies (Figure 4B, *p* = 0.012). Hypoactivity was particularly evident during the later portions of the test (∼60–90 min), suggesting that HET mice might have an increased rate of habituation to the testing environment. Analysis of thigmotaxis revealed that HET mice spent more time in the periphery rather than the center (Figure 4C, *p*<0.01), suggesting possible increases in anxiety in HET mice.

**Figure 5:**
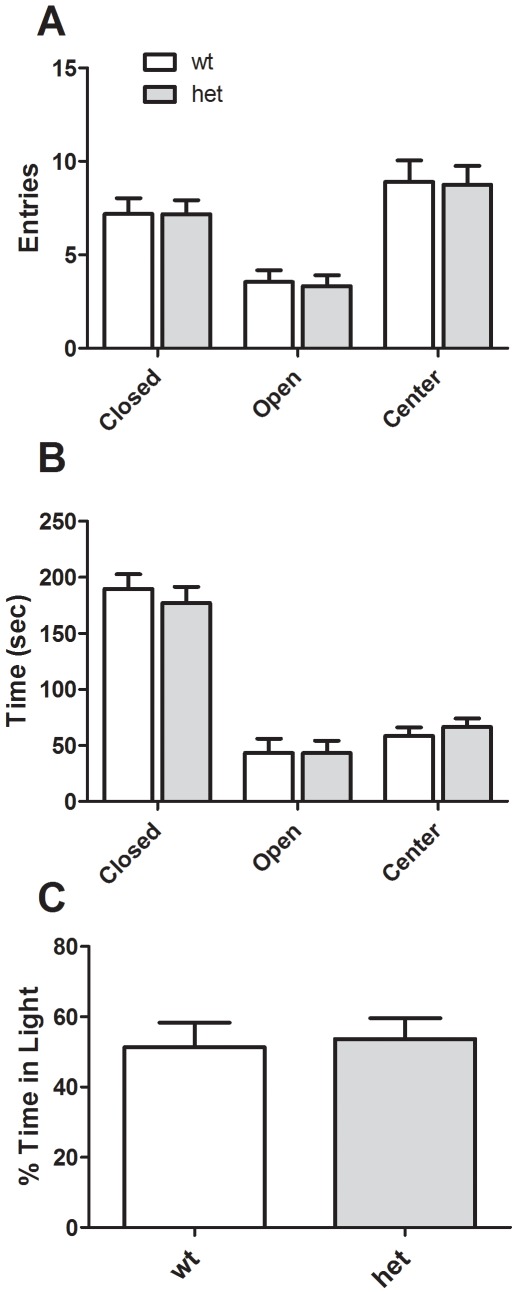
Anxiety-related behaviors in *CHIP* HET mice. Neither the number of arm entries (A) nor time spent in arms (B) in an elevated plus maze demonstrates differences in anxiety responses. Similarly, there are no differences between *CHIP* WT and HET mice in a light-dark preference assay (C).

We attempted to confirm an anxiogenic phenotype in *CHIP* HET mice using additional assays. In an elevated plus maze there was no significant effect of genotype on open arm entries [*F* (1, 22) = 0.035, p = 0.853] or time [*F* (1, 22) = 0.025, p = 0.874] (Figures 5A and B, respectively). As expected, there were significant effects of arm location, with more entries to the center and closed arms [*F* (2, 67) = 21.3, p<0.001] and a strong time preference for closed arms [*F* (2, 67) = 87.7, *p*<0.001]. We also measured protected and unprotected head dips and found no effect of genotype (data not shown). Finally, light-dark preference was unchanged (*p* = 0.803; Figure 5C), as was the latency to enter the dark zone for the first time (data not shown, *p* = 0.742).

**Figure 6:**
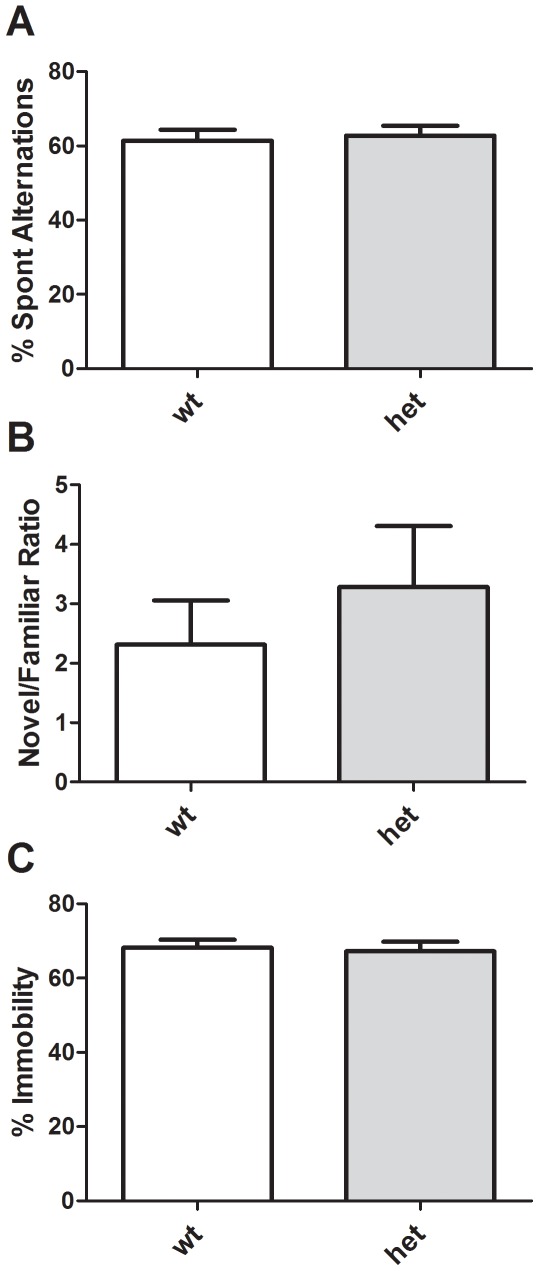
Cognitive and depressive behaviors in *CHIP* HET mice. No differences between *CHIP* WT and HET mice were noted in Y maze spontaneous alternation (A), novel object recognition (B) or tail suspension test (C) assays.

Spatial working memory was studied using spontaneous alternation in a Y maze. There were no significant differences in total arm entries (data not shown), or percent alternation (Figure 6A, *p* = 0.744). Nonspatial memory was assessed using a novel-object recognition paradigm and again no differences between genotypes were noted (Figure 6B, *p* = 0.450). Finally, mice were assessed for behavioral despair using the tail suspension test (Figure 6C), and no significant differences in immobility between genotypes were noted (*p* = 0.787).

Taken together, these studies indicate modest impairments in motor function, cardiovascular function and possibly anxiety status in *CHIP* haploinsufficient mice.

## Discussion

The E3 ubiquitin ligase CHIP binds to HSC70 and HSP70 and critically controls expression of both HSP70 and other essential client proteins, a feature which is more prominent when CHIP expression increases as a consequence of acute injury [15], [17], [37], [38], [39]. *CHIP* knockout mice are increasingly being used to examine neuroprotection and the molecular control of cell fate [17], [18], [19]. However, *CHIP* null mice die prematurely, with significant mortality observed in the peripartum and early postnatal periods, and exhibit a host of severe neurological and peripheral phenotypes [19], including profound ataxia and growth impairments. In contrast, a single *CHIP* allele disruption results in a 50% loss of CHIP expression, but no overt phenotype [19]. Use of heterozygotic mutants allows for clearer interpretation of abnormal phenotypes, and likely a better translation to human disease given that, at least at this time, no total loss-of-function mutations in CHIP have been observed in human disease states. In the present study, therefore, we conducted a battery of neurobehavioral and physiological experiments to provide a better understanding of the functional consequences of *CHIP* haploinsufficiency.

*CHIP* HET mice exhibited normal survival, growth rates, body temperature, grooming, muscle tone and breathing patterns. There was a small, but statistically significant elevation of heart rate in the mutant mice. As heart rate was measured in awake mice, it is possible that the increase in heart rate is a physiological manifestation of behavioral stress. Alternatively, this may indicate an intermediate phenotype to the significant cardiac hypertrophy observed in *CHIP* knockout mice. Somatosensory responses, grip strength, pole climbing and coordination (i.e. rotarod performance) were also normal, but some aspects of motor control were deficient in the *CHIP* HET animals. The haploinsufficient mice fell more quickly from both a wire and an inverted screen and had difficulty using their hind paws in a wire maneuver task.

When placed in a novel open field, *CHIP* HET mice exhibited significantly less motor stereotypies than control animals and traversed less horizontal distance specifically in the center of the arena. Thus, the *CHIP* mutants spent more time near the edges of the chamber, rather than in the more open center portion. This phenotype is generally thought to reflect an increase in anxiety state, although there are other possible explanations [40]. We also noted that the *CHIP* HET mice were more timid during routine handling procedures reflected by an increased startle response (unpublished observations) [41], which was surprising given that when animals were examined in the elevated plus maze and light-dark tasks, no alterations in anxiety behaviors were apparent. Expression of behavioral despair in a tail suspension assay was also normal. Taken together, these observations suggest that *CHIP* HET mice may express subtle context specific alterations in anxiety and/or stress reactivity that were not captured by our testing. Future studies using additional testing paradigms and neuroendocrine measures will be necessary to explore this further.

We employed spontaneous alternation in a Y maze as a basic screen for spatial working memory [32], [33], but observed no significant changes. Rates of spontaneous alternation are also influenced by attention, anxiety and arousal state (for reviews, see [42], [43]). Similarly, a novel object recognition paradigm was used to explore short-term nonspatial working memory [32], [33], but no alterations were noted. Long-term learning and/or memory may be affected in the mutant mice, but has not been examined at this time. It is also worth noting that all of our studies were conducted in young adult mice; it is possible that *CHIP* mutant mice exhibit premature age-related declines in performance in these tasks.

Mice heterozygotic for another E3 ubiquitin ligase *Scrapper*, have also recently been behaviorally characterized [27] and it is worth noting some parallels between these mice and *CHIP* haploinsufficient mice. Scrapper is one of a group of E3 ligases with essential roles in neurite outgrowth. The protein is localized to synapses and *Scrapper* null mice die at birth. The heterozygous mice are viable and grossly normal. Detailed analyses suggest normal motor function and anxiety level through elevated plus maze and light-dark tests; however, *Scrapper* HET mice show changes in center-surround activity patterns in an open field, with increased center time [27]. In the current study, we again see context-specific effects on anxiety measures, but *CHIP* HET mice exhibit increased anxiety in an open field. Similar to the CHIP mutants, *Scrapper* HET mice also showed normal sensory responses and were not different from controls in most learning and memory paradigms. *Scrapper* HET mice, however, did exhibit impaired contextual fear conditioning (but not cue) [27], suggesting deficits in some aspects of hippocampal function. It will be very interesting to assess fear conditioning in *CHIP* HET mice in the future. Social approach and aggression would also be interesting domains to explore [44], [45], [46].

It has been hypothesized that protein dysregulation and increased accumulation of dysfunctional organelles, as well as proteins, result in late-onset neurodegeneration [15], [47]. Mice with a *full* deletion of the E3 ligase Parkin have remarkably subtle pathological and behavioral changes [48], [49], although mutations in the PARK2 gene, coding for Parkin, constitute the most common familial form of PD [50]. CHIP overexpression compensates for loss of Parkin activity suggesting that these proteins have homologous functions and client proteins. Our data taken with that of our colleague Dr. Patterson, who initially characterized this mutant mouse, suggest that CHIP plays a more central role in CNS and cardiac cell stress and survival. Given that CHIP function and expression in the brain decline during aging suggests that decreased levels of CHIP are sufficient to induce dysfunctions that might be associated to human neurodegenerative diseases. Once we move CHIP deficiency from the hybrid B6/C57 to their 10^th^ generation of breeding on the B6 background, we will confirm these behavioral traits and pursue analysis of CNS pathology in homozygote and heterozygote animals [51], [52].

In summary, our data point to the presence of specific motor disturbances in *CHIP* HET mice, suggesting that moderate underexpression of CHIP produces functional impairments in brain circuits. In contrast, we observed only a very modest anxiety phenotype and no changes in performance in simple tasks of behavioral despair and learning and memory. Taken together, these data indicate a relatively subtle but clear phenotype of *CHIP* haploinsufficiency which will help guide future studies of the effects of CHIP dysregulation on brain circuitry, function, and neuronal responsiveness to environmental and cellular stress.
